# Nitrogen Exchanges: Testing the Hypothesis of a Country without Agricultural Production

**DOI:** 10.1100/tsw.2001.306

**Published:** 2001-11-07

**Authors:** M.-F. Slak, L. Commagnac, P. Pointereau, S. Larbouret, C. Lucas, S. Muller

**Affiliations:** Ecole Nationale d'Ingénieurs des Travaux Agricoles de Bordeaux, Laboratoire Sols et Paysages, Gradignan, France

**Keywords:** nitrogen balance, agriculture, society

## Abstract

Today, finding data on agricultural nitrogen balances is quite easy. Calculations of such balances are carried out by most of the European countries as an indicator of environmental pollution attributable to the agricultural sector. In France, average values of agricultural nitrogen balances show an excess of 1.5 to 2 million tons of nitrogen. This excess is enormous. What would the balance of a country be if agricultural activity were stopped? In the following article, a country (France is used as an example) without agriculture is studied in order to assess its nitrogen balance. Using a previously published model describing nitrogen input and output of a given country, nitrogen flows are identified. Inputs include deposition, fixation, and products not intended for agricultural use. Outputs are reduced to zero if agriculture disappears (in France, agriculture is the only sector exporting products containing nitrogen). All flows are calculated considering the hypothesis of disappearance of agriculture. Nitrogen requirements to feed people and pets in France are estimated based on medical and veterinary data (recommended daily amounts for proteins and/or usual average consumption). Indeed, most of the food that nourishes the French population is produced nationally. If agriculture stops, it will be necessary to import food from foreign countries. Results show an unexpectedly high excess (for a country without agriculture having a structure similar to France: number of human beings and pets) of 1.5 million tons of nitrogen. An attempt to calculate an agricultural balance with the same data gives a result close to 3 million tons. Differences in French agricultural balances found in the literature can mainly be explained by values taken into account for deposition and fixation (values used here are at least 300,000 tons higher than values used by the Organisation for Economic Co-operation and Development). In conclusion, nitrogen excess in agriculture is partly due to social demand; agriculture does not only produce food but also includes many other functions (landscape management, employment, and preservation of culture, for example). As a consequence, efforts that do not involve suppressing agriculture should be made to figure out alternative ways of production.

### INTRODUCTION

Water pollution by nitrates is a major concern worldwide and in France in particular because of the importance of the agricultural sector and the intensive methods of production used (the latter is particularly true in some areas such as Brittany). Agriculture is responsible for a great share of nitrogen losses; hence agricultural nitrogen balances are used as indicators of environmental nitrogen pollution by the agricultural sector. Regardless of which method is used to calculate these balances, results show a large excess of nitrogen in this sector (estimates range from 1.5 to 2 million tons of surplus of nitrogen). What would the balance of a country without an agricultural sector be? Would it be equal to zero?

Having worked for several years on nitrogen balances in European countries using international trade data[1,2], we considered the case of a country without agriculture. In this article the hypothesis of the disappearance of agriculture is applied to a country having the structure of France (e.g., number of inhabitants, number of pets, area) in order to assess the nitrogen balance of such a country. First, a study of the effect of the disappearance of agriculture on the input and the output flows is carried out, then an attempt to quantify these flows is made.

### MATERIALS AND METHODS

Clearly, as hypothetical country X, based on French data, will import foodstuff, a nitrogen excess will necessarily occur. Inputs of nitrogen-containing products (e.g., fertilizers) will decrease but, at the same time, outputs will also decrease because agriculture is the main sector exporting nitrogen-containing products (raw food products both for animals and human beings). Using the model of national farm gate balance established[1,2], Fig. 1 shows the impact of the disappearance of agriculture on nitrogen flows.

French nitrogen input through fishing activities was estimated, but because it is quite low (10,000 tons of nitrogen) this inflow was not taken into account.

In the case of the disappearance of agriculture, many of the flows represented in Fig. 1 will vary greatly. Imports intended for the agricultural sector will be reduced to zero (such imports generally represent a considerable path of input for nitrogen through livestock feed and fertilizers) as well as exports from the agricultural sectors.

All products currently obtained from the national agriculture production for feeding people will need to be imported as well as those intended for feeding "nonagricultural" animals such as pets, horses, and zoo animals. Modifications of nitrogen flows can be described as follows.

### Inputs

1. *Atmospheric nitrogen deposit* value may decrease a few years after agricultural production stops because organic manure is a high source of gaseous ammonia. Atmospheric currents redistribute gases between areas but this phenomenon occurs only on small scale.
2. *Fixation* will still exist, but the value of the flow itself will be modified. There will no longer be cultivated leguminous crops; however, legumes are found in natural pastures at a rate of about 20%, at least in France. Nevertheless, it is more likely that forests will grow on derelict agricultural land; thus, the value of nitrogen fixed by legumes will decrease.
3. Results obtained earlier allow us to assess nitrogen *input and output flows due to nonagricultural activities* (e.g., leather for clothes, nitrous acid, or powder for ammunitions). These calculations are based on our previous work (nitrogen balances are estimated using data from trade exchanges [1,2,12] which was carried out on the entire French territory, the agricultural sector being included.)

Most of the input and output flows resulting from nonagricultural activities are due to chemicals or manufactured products that will continue to be imported. If agriculture stops, the value of the input flow of nitrogen products not intended for agricultural use will increase. This augmentation will mainly be due to the increased amount of food imported. This augmentation will

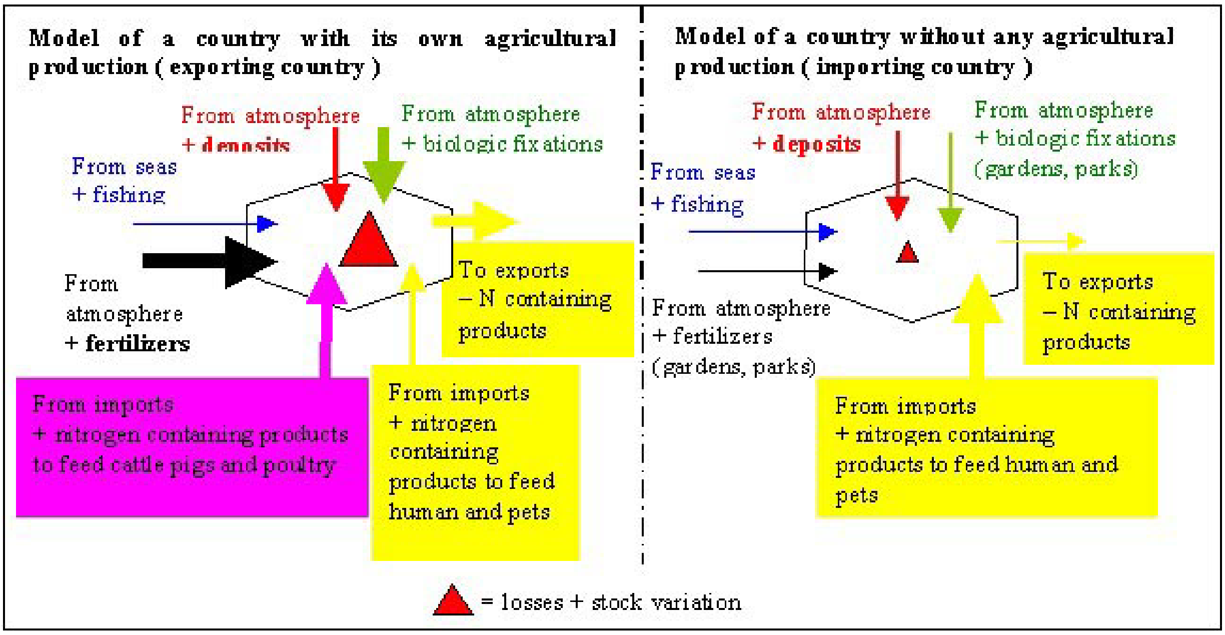

**FIGURE 1**. Comparisons of nitrogen flows with and without agriculture. The left side of the figure represents the current situation. The national nitrogen balance is the difference between inputs and outputs. The discrepancy is made up by stock variations and losses. Variations in arrow width represent main flows that would be modified by the disappearance of agriculture: deposits from atmosphere, biological fixations, fertilizer imports, food imports and exports of nitrogen-containing products.

simultaneously be found to equal the amount of nitrogen in the food now produced by French national agriculture.

Quantification of the amount of nitrogen necessary to feed the population and its pets in a country having the structure of France has been estimated using a method described below.

### Outputs

Agricultural production being null, the only exports of nitrogen-containing products would occur through chemical products. As France is a net-importing country for chemicals and man-made products, exports of nitrogen-containing products would be zero.

To calculate the nitrogen balance of such an imaginary country, the only flow still to be estimated is the import of food products for human beings and pets necessary to nourish the population of this dependent country. Databases with recommended daily amounts of proteins can be found in the literature both for human beings and pets. These amounts depend on age and sex. For humans, data on population structure have been obtained from the French census of 1999[3].

As far as pets are concerned the problem is more complex. Recommended daily amounts of protein are expressed in grams per kilogram of body weight; thus, the number of "kilograms of living dogs and cats" was estimated. To calculate the number of pets, the results of a sample survey carried out at the request of the association of processed feed producers for dogs, cats, birds, and other pets[4] were used. The survey concerned dogs and cats only and indicates population structure (breeds) and quantity. The average weight of a dog (average weight of the ten most popular breeds) was multiplied by the number of dogs. The same was done with cats, considering an average cat weight of 4.5 kg. Once protein requirements were identified, a ratio of 6.25 was applied to obtain a value in kilograms of nitrogen.

No estimation was done here regarding horses and exotic animals, so needs are underestimated.

## RESULTS

As mentioned earlier, the emission of nitrogen to the atmosphere from the agricultural sector is mainly due to organic manure. If the agricultural sector disappears, there will not be any manure production. As a consequence, atmospheric nitrogen deposit should decrease by the amount of ammonia losses that occur during manure storage and spreading. Today manure production represents 1.18 million tons of nitrogen[5]. Total losses of nitrogen through volatilization due to agricultural practices represent 789,200 tons of ammonia (i.e., 650,000 tons of nitrogen) for 1998[6]. Other sources of nitrogen due to agriculture include $NO_x$, which totals 236,900 tons (i.e., 90,300 tons of nitrogen), and $N_2O$, which totals 194,500 tons (123,700 tons of nitrogen). These emissions are not produced by manure. The sum of all emissions (total emissions of ammonia plus total emissions of $NO_x$ plus total emissions of $N_2O$) is 1,497.7 expressed in thousands of tons of nitrogen (of which 864,000 tons are from agricultural sources). For the year 1997, the Co-operative Programme for Monitoring and Evaluation of the Long Range Transmission of Air Pollutants in Europe (EMEP)[7] gives the following figures: total emissions for France were 1.05 million tons of nitrogen while total deposition was 747,000 tons of nitrogen. An average of the ratio "total deposition divided by total emission" calculated over 13 years with EMEP data gives a result of 61%. If we apply this ratio to agricultural emissions, deposition should drop to 527,000 tons of nitrogen. Deposition calculated with the same method from Inter-professional Technical Centre for the Study of Atmospheric Pollution[6] data gives a total deposition of 850,000 tons of nitrogen for the year 1998. This result means that agriculture could be responsible for 62% of nitrogen emissions.

Regarding fixation, it is possible to estimate a value corresponding to the following hypothesis: if agriculture disappears, leguminous plants will be present only on natural pastures. To estimate fixation, an average value for nitrogen fixed per hectare for each of the main leguminous crops grown in France was used. Values for fixation were found in literature[8] as well as the areas of the various crops. Table 1 summarizes these results.

**TABLE 1**
**Fixation by Main Leguminous Plants in France**

| Legumes | Area (ha) | Mean Fixation (kg/ha/year of N) | Total Fixation (tons of N) |
|---|---|---|---|
| Bean | 13,309 | 210 | 2795 |
| Haricot bean | 51,725 | 70 | 3621 |
| Lentil | 6018 | 101 | 608 |
| Lupine | 4890 | 176 | 861 |
| Alfalfa | 358,118 | 200 | 71,624 |
| Pea | 35,311 | 65 | 2295 |
| Soya | 102,079 | 75 | 7656 |
| Clover | 44,993 | 183 | 8234 |
| Mix | 34,051 | 186 | 6345 |
| **Total** | **650,494** | | **104,039** |

*Note:* Areas are from [9] and values for fixation are based on [8]).

To the amount of nitrogen fixed by cultivated leguminous crops, the amount of nitrogen fixed by leguminous plants in natural pastures must be added. In France, it is generally considered that natural pastures contain about 20% leguminous plants. French natural pastures cover 7 million ha, which is equivalent to 1.4 million ha of cultivated leguminous crops in terms of fixation using the above ratio (one fifth). For these 1.4 million ha, the amount of nitrogen fixed per hectare could be estimated by an average of fodder legumes fixation: alfalfa, clover, and lupine. This calculation gives an average fixation of 186 kg/ha/year. Applied on the theoretical surface of 1.4 million ha, the result is about 261,000 tons of nitrogen fixed by leguminous plants in natural pastures. Of course, this value supposes that most of the natural pastures will be maintained for the feed of animals kept for leisure (e.g., horses). It seems logical to consider that no mineral fertilizers will be applied on these areas; therefore, symbiotic fixation by leguminous plants should be quite high.

The sum of cultivated leguminous crops fixation and nitrogen fixed by legumes in natural pastures gives a result of 365,000 tons of nitrogen fixed per year. Without agricultural production, leguminous crops will disappear, so the amount of nitrogen fixation due to cultivated leguminous crops (104,000 tons of nitrogen) must be subtracted. The result of 261,000 tons may overestimate fixation because areas in natural pastures could diminish (fewer animals to feed).

Imports of nonagricultural products (e.g., chemicals and man-made products) have been estimated using a method described in Slak et al.[1,2]. Basically, the method consists of applying a nitrogen-content value to each commercial product exchanged between a given country and any other country of the world. This value applies to agricultural products as well as to nonagricultural products. Using this method, it was found that France is a net importer of 517,000 tons of nitrogen coming from chemicals and man-made products. Because these products are mainly intended for industry, this flow will not change a lot if agriculture disappears; for this reason the same value was kept.

The last flow to estimate is the amount of nitrogen necessary to feed people and pets. Calculations are based on recommended daily amounts (RDAs) for protein (average needs plus two standard deviations)[10]. First, an assessment was made of the volume of nitrogen that should be imported to feed the population. France has a population of 58 million people[3]. Protein RDA varies from individual to individual and depends mainly on age and sex. Table 2 comes from the French National Institute of Statistics[3] and gives a picture of the structure of the French population in 1999.

Several sources give protein RDA, including the Food and Agriculture Organization of the United Nations (FAO) and the World Health Organization[10]. Unfortunately, there are as many values as there are sources. To carry out calculations, RDAs from a report of the French Institute for Health and Medical Research[8] were used. Average values of protein RDA in grams per kilogram per day for children and adults are given based on FAO recommendations. Table 3 shows these values.

If there were no agriculture, France would have to import 335,500 tons of nitrogen to feed its population. Of course nitrogen imports would be much higher because many food products contain nitrogen in inedible parts (e.g., skin, stone, or bones), and there is always some food that goes to waste (e.g., meal scraps, spoiled products). Average amounts of wastes produced by food products has not yet been assessed; thus, the value of 335,000 tons of nitrogen will be used as an estimation of the amount of nitrogen in the food intended for the French population.

Calculations must include pets. As mentioned earlier, horses and small or exotic animals (e.g., rabbits, birds, fishes, or snakes) will not be taken into account; we will focus on cats and dogs. It is necessary to underline the fact that the number of cats and dogs in France is the highest in Europe: there are 8.1 million dogs and 9 million cats[4]. Protein RDA is generally expressed in grams per kilogram of body weight. Average weights of the ten most popular dog breeds were supplied by a veterinarian and were used to calculate an average weight for dogs, which is about 21.3 kg. Multiplying this value by the number of dogs gives a number of kilograms of living dogs. Using a value of 5 g/kg/day for protein RDA for both dogs and cats, 50,000 tons of nitrogen would be needed each year to feed dogs (number of dogs $\times$ average dog weight $\times$ RDA $\times$ 365)/6.25. The same calculation for cats (with an average weight of 4.5 kg) gives a result of 11,826 tons of nitrogen.

Calculations are based on *protein consumption*; thus, even if nowadays cats and dogs eat a lot of meal scraps, this part of their diet is not included in the 335,500 tons of nitrogen needed to feed humans. As a consequence, to feed pets, 60,000 tons of nitrogen would be needed if agriculture disappeared in France.

**TABLE 2**
**Results of the French Census of 1999[3]**

| Age Group | Men | Women | Total |
|---|---|---|---|
| 0 – 1 year | 433,251 | 413,709 | 846,960 |
| 2 – 3 years | 731,913 | 695,250 | 1,427,163 |
| 4 – 6 years | 1,079,567 | 1,030,351 | 2,109,918 |
| 7 – 10 years | 1,532,291 | 1,460,766 | 2,993,057 |
| 11 – 14 years | 1,571,031 | 1,501,210 | 3,072,241 |
| 15 – 49 years | 14,430,462 | 14,435,892 | 28,866,354 |
| > 50 years | 8,640,904 | 10,564,091 | 19,204,995 |
| **Total** | **28,419,419** | **30,101,269** | **58,520,688** |

**TABLE 3**
**RDAs and Real Protein Consumption by Group of Age**

| Age Group | RDA (g/person/day)[1] | Real Protein Consumption[2] | Number | Estimated Consumption (tons of nitrogen/year)[3] |
|---|---|---|---|---|
| 0 – 1 year | 14 | 14 | 846,960 | 692 |
| 2 – 3 years | 17.5 | 47 | 1,427,163 | 3917 |
| 4 – 6 years | 24.75 | 63 | 2,109,918 | 7762 |
| 7 – 10 years | 31 | 80 | 2,993,057 | 13,983 |
| 11 – 14 years | 42.25 | 80 | 3,072,241 | 14,353 |
| 15 – 49 years | 55.5 | 105 | 28,866,354 | 177,008 |
| > 50 years | 53.75 | 105 | 19,204,995 | 117,765 |
| **Total** | | | **58,520,688** | **35,3480** |

*Note*: Nitrogen equivalent estimation.

[1] Values are FAO guidelines: FAO, 1987.
[2] Values based on a report of the French Institute for Health and Medical Research[9].
[3] Result was calculated as follows: (real protein consumption × number of people in age group × 365)/(6.25 × 1,000,000); proteins contain about 16% nitrogen; for this reason a ratio of 6.25 was used.

Finally, if agriculture disappeared in France, 400,000 tons or so of nitrogen would be needed to feed the French population and its pets, based on their protein consumption. Real imports would be even higher because of the difference between nitrogen imported and nitrogen present in edible parts of food products.

All flows of nitrogen arriving in or leaving a country having a structure like that of France have been assessed; thus, it is now possible to calculate the nitrogen balance for such a hypothetical country without agriculture. Because France is a net importer of chemicals or manufactured products, there will be no output. To calculate the input, total deposition (261,000 tons of nitrogen), total fixation (322,000 tons of nitrogen), total imports due to nonagricultural products (517,000 tons of nitrogen) and food imports for pets and humans (400,000 tons of nitrogen) must be added.

*The result is a positive balance of 1.5 million tons.* The Organisation for Economic Co-operation and Development (OECD) agricultural nitrogen balance gives a number of 1.5 million tons of nitrogen in excess for the agricultural sector. It is necessary to underline here the fact that the OECD calculations were carried out on a soil surface balance model (i.e., the system studied is limited to the soil of the farms) applied at a national scale while our model uses a model similar to the “farm gate balance”, considering the system studied (the national farm) as a black box. Table 4 summarizes results obtained and attempts to compare these results with the OECD balance.

Of course, the difference between the agricultural balance and the “nonagricultural” one is closer to 1.7 million tons of nitrogen than to 1.2 million tons of nitrogen (because of the 500,000 tons of chemicals and manufactured products imported). The excess calculated using the above method for the agricultural sector is much higher than the one calculated by the OECD. Table 5 allows a more precise comparison between the OECD nitrogen agricultural balance and the agricultural balance derived from the above calculations.

Two factors help to explain this discrepancy.

- The different methods do not take into account the same input and output; the OECD balance *is a soil surface balance*, whereas the method used here is closer to a “farm gate” balance; the latter often gives higher results.
- Values for fixation and deposition vary a lot depending on sources.

Considering the excess of 3 million tons of nitrogen for agriculture, it can be assumed that the agricultural sector is “responsible” for 2 million and society for the remaining 1 million (1 million tons of nitrogen = balance without agriculture [1.5] minus chemical and manufactured products which are not taken into account in balance n$^{o}$2[0.5]). Indeed, as shown in this article, even if there were no agricultural sector, *the balance would still be quite high if satisfying the population’s needs continued to be a priority*.

### CONCLUSION

The value of the nitrogen balance of a country without agriculture is far from being low. When agriculture is present, the value of the balance is much higher (about 1.7 million tons of nitrogen higher). It should be stressed that even if agricultural production is too high in Europe today and generates food surpluses for export, it is impossible to consider a real disappearance of agriculture. Not only would such a disappearance affect employment (the food industry is very large in France), but it would also affect tourism. The food industry and tourism are, respectively, the first and second positive line items in the French balance of foreign trade. Without agriculture, the landscape would dramatically change, and this change probably would have a negative impact on tourism.

**TABLE 4**
**Summary of Results Obtained and Attempts to Calculate an Agricultural Balance**

| Balance | Flow | Deposition | Fixation | Chemicals and Manufactured Products | Food | Fertilizers | Feed for Stock Farming | Products Produced by Agriculture[1] | Total |
|---|---|---|---|---|---|---|---|---|---|
| 1. Without agriculture | Input | 323 | 261 | 517 | 400 | 0 | 0 | 0 | 1501 |
| | Output | 0 | 0 | 0 | 0 | 0 | 0 | 0 | 0 |
| | Balance | 323 | 261 | 517 | 400 | 0 | 0 | 0 | 1501 |
| Hypothesis | Value = | Total deposition | Total fixation | 0[2] | 0[3] | Current use[4] | Current imports[5] | Current production[5] | |
| 2. With agriculture in order to allow comparison with OECD | Input | 850 | 365 | 0 | 0 | 2500 | 400 | 0 | 4115 |
| | Output | 0 | 0 | 0 | 0 | 0 | 0[3] | 1400 | 1400 |
| | Balance | 850 | 365 | 0 | 0 | 2500 | 400 | –1400 | 2715 |

*Note*: All values are in thousands of tons of nitrogen.

1 The agricultural model used here considers all raw products arriving in or leaving from the “national farm”. As a consequence, the item “Products produced by agriculture” includes food products (vegetables) and products intended for feeding stock farming animals (cereals) as well as products intended for other purposes.

2 Because the OECD balance is an agricultural balance, chemicals and manufactured products not intended for agriculture must not be taken into account. It is clear that this point is debatable because regardless of which balance is taken into account, these products are still imported.

3 The agricultural sector being active, food for the population is produced nationally (in France, the national agricultural sector produces most of the food needed by the population), and there is no need to import it.

4 Data are from [11].

5 Values are from calculations carried out in previous work[1,2].

**TABLE 5**
**Attempt to Compare Balance 2 from Table 4 with the OECD Agricultural Balance**

| | Mineral Fertilizers | Organic Manure | Deposition + Fixation | Plant Harvests | Grazing | Feed for Stock Farming | Products Produced by Agriculture | Balance |
|---|---|---|---|---|---|---|---|---|
| OECD balance [10] | 2457 | 1183 | 910 | –1986.5 | –978.5 | — | — | 1585 |
| Balance (2) from the above table | 2500 | — | 1215 | — | — | 400 | –1400 | 2715 |
| Difference | –43 | 1183 | –305 | — | — | — | — | –1130 |

*Note:* All values are in thousands of tons of nitrogen.

The liberty of choice regarding cultivation of transgenic crops as well as the assurance of food independence are major demands of society; agriculture is able to meet these demands and must do so. Agriculture also fulfils many other functions regarding society such as landscape management, the relationship between humans and nature, and protection of culture (this last is especially true for France with its numerous local cheeses and regional dishes).

The agricultural sector, as managed nowadays, generates a high nitrogen excess (another paper presents alternative ways of managing nitrogen resources). By calculating agricultural balances only, we mask the fact that agricultural nitrogen surpluses are partly due to consumer and society demand.

### ACKNOWLEDGEMENTS

My staff and I would like to thank EUROSTAT for their kindness and for the help they provided us to carry out this project. I also want to thank Lee Valent who corrected the final version of this manuscript.

| | | |
|---|---|---|
| Received: | July | 27, 2001 |
| Revised: | October | 8, 2001 |
| Accepted: | October | 15, 2001 |
| Published: | November | 7, 2001 |
